# Association of dietary approaches to stop hypertension eating style and risk of sarcopenia

**DOI:** 10.1038/s41598-020-76452-0

**Published:** 2020-11-09

**Authors:** Sanaz Soltani, Rezvan Hashemi, Ramin Heshmat, Ahmadreza Dorosty Motlagh, Ahmad Esmaillzadeh

**Affiliations:** 1 Department of Community Nutrition, School of Nutritional Sciences and Dietetics, Tehran University of Medical Sciences, P.O. Box 14155-6117, Tehran, Iran; 2 Department of Geriatric Medicine, Ziaeian Hospital, Tehran University of Medical Sciences, Tehran, Iran; 3 Chronic Diseases Research Center (CDRC), Endocrinology and Metabolism Population Sciences Institute, Tehran University of Medical Sciences, Tehran, Iran; 4 Obesity and Eating Habits Research Center, Endocrinology and Metabolism Molecular-Cellular Sciences Institute, Tehran University of Medical Sciences, Tehran, Iran; 5 Department of Community Nutrition, Isfahan University of Medical Sciences, Isfahan, Iran

**Keywords:** Diseases, Geriatrics, Nutrition

## Abstract

The association between habitual intake of the “dietary approaches to stop hypertension” (DASH) eating plan and sarcopenia has received limited attention. The present study aimed to investigate the association between adherence to DASH dietary pattern and sarcopenia and its components including muscle mass, muscle strength, and muscle performance among community-dwelling older adults population. This population-based cross-sectional study was performed in 2011 among 300 older people (150 men and 150 women) aged ≥ 55 years, who were selected using cluster random sampling method. Dietary intake of study participants were examined by the use of a Block-format 117-item food frequency questionnaire (FFQ). The DASH score was constructed based on eight main foods and nutrients emphasized or minimized in the DASH diet. All components of sarcopenia was measured using standard protocols and sarcopenia was defined based on both former and new European Working Group on Sarcopenia in Older People (EWGSOP) guidelines. Mean age and BMI of study participants were 66.7 ± 7.7 years and 27.3 ± 4.2 kg/m^2^, respectively. Totally, 31 individuals meet the criteria of EWGSOP2-sarcopenia. We found no significant association between adherence to the DASH diet and EWGSOP2-sarcopenia either before (OR 1.08; 95% CI 0.45–2.54) or after adjustment for potential confounders (OR 1.04; 95% CI 0.39–2.75). The same findings were obtained in the gender-stratified analyses (men: OR 2.29; 95% CI 0.39–13.29 and women: 0.75; 95% CI 0.23–2.45). In conclusion, we found that adherence to the DASH-style diet was not significantly associated with odds of sarcopenia. Future prospective studies are required to confirm these findings.

## Introduction

Sarcopenia as a geriatric syndrome^1^ is a concept that describes the loss of skeletal muscle mass and strength^2^. This condition is associated with the risk of physical frailty, functional impairment, poor quality of life, and premature death in older individuals^3,4^. Interest in sarcopenia identification has grown since its recent recognition as a muscle disease with an International Classification of Diseases, Tenth Revision, Clinical Modification (ICD-10-CM) diagnosis code^5^. Sarcopenia wastes a significant cost in the healthcare systems. In 2000, about $18.5 billion (1.5% of total healthcare expenditures) was spent in the United States on healthcare expenses related to sarcopenia and its complications^6^. In Iran, there is no information on the costs that sarcopenia imposes on the healthcare system. The prevalence of this disorder varies largely based on the definition or cut-off value across studies^7^. Accordingly, the prevalence of sarcopenia varies worldwide from 9.9 to 40.4%^8^.

Healthy eating is highly recommended to preserve physical health in later life. Poor diets and nutritional status are common among older people^9,10^. The Dietary Approaches to Stop Hypertension (DASH) eating plan, as a healthy dietary pattern, characterized by low intake of saturated fat, meats, sweets, added sugars, and sugar-sweetened beverages and high intakes of fruit and vegetables, low-fat dairy, whole-grain products, nuts, fish and poultry^11^. This dietary pattern has been protectively linked with several conditions such as cardiovascular disease^12^, cancer^13^, type 2 diabetes^14^; however, limited data are available examining adherence to this dietary pattern in relation to sarcopenia. Although usual nutritional advices to prevent sarcopenia include protein or amino acids intake, the DASH diet, through its myo-protective components^15–19^, might also play a direct or indirect role in maintaining muscle health. Adherence to DASH diet may directly affect muscle health by ameliorating metabolic processes (like oxidative stress, inflammation and insulin resistance) involved in sarcopenia^20–27^. In addition, DASH diet may reduce the risk of age-related chronic conditions (e.g., cardiovascular diseases, diabetes and cancer), that are associated with worsening muscle health^12,14,28–31^. We are aware of only two studies that examined the link between DASH diet and sarcopenia or its components^32,33^. Adherence to this eating style in African American and White adults was associated with better handgrip strength (HS), which is a simple and reliable method to evaluate muscle function^32^. However, the study of Kuczmarski et al. examined the association of DASH diet with handgrip strength alone, not the sarcopenia^32^. In a quasi-experimental study by Straight et al., administration of DASH diet for eight weeks in overweight and obese older adults resulted in improved body composition, muscle strength, and physical function^33^. Although findings from both observational and interventional studies are needed to establish the association between DASH diet and sarcopenia, findings from interventional studies cannot easily be extrapolated to human routine life. Interventional studies might be appropriate to detect the effect of an intervention on sarcopenic conditions. In order to assess if adherence to the DASH diet can prevent sarcopenia, it seems that observational studies are required. Therefore the present study aimed to investigate the association between adherence to DASH dietary pattern and sarcopenia and its components including muscle mass, muscle strength, and muscle performance among community-dwelling older adults population.

## Materials and methods

### Participants

This population-based cross-sectional study was performed from May to October of 2011 in Tehran, Iran (the detailed report on the sampling method and data collection procedure, have been published previously^34^). We enrolled 300 older people (150 men and 150 women) aged ≥ 55 years from district 6 of Tehran using cluster random sampling method. We selected the head of each 30 cluster based on a ten-digit postal code and enrolled individuals aged ≥ 55 years, with the ability to move without crutches, walker or assistive devices and those without any active cancers (based on self-reported data). We did not include people who were susceptible to sarcopenia including individuals with artificial limbs or limb prosthesis and those with a history of debilitating disease including Congestive Heart Failure (CHF), Chronic Obstructive Pulmonary Disorder (COPD), Chronic Renal Failure (CRF), cirrhosis and liver failure (based on self-reported data).

The study was conducted according to the guidelines of Declaration of Helsinki. The study protocol was approved by the Tehran University of Medical Sciences ethics committee. At first, participants were briefly informed about the objectives of the survey. All participants completed the written informed consent before data collection. Then all required data were collected through face-to-face method by a trained interviewer at home.

### Dietary assessments

Dietary intake of study participants were examined by the use of a Block-format 117-item food frequency questionnaire (FFQ). The questionnaire included a list of food items, along with a given portion size and an open-ended frequency response section. A trained nutritionist administered the FFQs. Participants were requested to report their daily, weekly or monthly frequency consumption of each food item in the questionnaire during the preceding year. Frequency data in the FFQ were then converted to grams per day considering the household measures of portion sizes. Then these data were linked to the modified food composition database of the US Department of Agriculture, using Nutritionist IV software (version 7.0; N-Squared Computing, Salem, OR, USA), to compute daily energy and nutrients intake of each participant. The seasonal variation in dietary intake of fruit and vegetables was considered in the questionnaire; such that participants were requested to report their consumption of specific fruit and vegetables during the time of availability on that item. Then, when we were computing the grams of food items, we considered duration of availability of that food item in a year in our calculation.

Although the FFQ was validated previously^35^, we conducted a pilot study on 30 participants to examine its validity in older adults’ population. To do this, nutrient intakes of the FFQ were compared with those obtained from four dietary records (two records in weekdays and two other records after 2 months). The results showed a good correlation between the dietary intakes determined by the FFQ and those from the self-reported records. The correlation coefficient for animal protein, fruits, and vegetables were 0.43, 0.57, and 0.45, respectively. The energy adjusted correlation coefficients for β-carotene and vitamin C were 0.65 and 0.76, respectively.

### Adherence to DASH-style diet

In this study, to indicate the degree of adherence to DASH-style diet, we constructed DASH scores based on foods and nutrients emphasized or minimized in the DASH diet, focusing on eight components: high intake of fruits, vegetables, nuts and legumes, low-fat dairy products, and whole grains and low intake of sodium, sugar-sweetened beverages (SSB) and sweets, and red and processed meats. For each participant, energy-adjusted intakes of the above-mentioned foods and nutrients were calculated using residual method^36^. Then, participants were categorized into deciles of the energy-adjusted intakes of foods and nutrients. Subjects in the highest decile of fruits, vegetables, whole grains, low-fat dairy products, nuts, legumes and soy were assigned the score of 10 and those in the lowest decile were assigned the score of 1. With regards to red and processed meat, sugar-sweetened beverages (SSB) and sweets, and sodium, we did vice versa; that is individuals in the highest decile were assigned the score of 1 and those in the lowest decile were assigned the score of 10. Then, the overall DASH score was calculated by summing up the scores of eight components. Therefore, each participant has a score between 8 and 80.

### Assessment of sarcopenia

We applied both former^37^ and new^38^ European Working Group on Sarcopenia in Older People (EWGSOP) guidelines for sarcopenia in our study. EWGSOP1 recommends considering the combination of both low muscle mass and low muscle function (either strength or performance) in the definition^37^. Based on EWGSOP2, low muscle strength is a key component of sarcopenia. In addition, this definition uses low muscle quantity and quality to confirm the diagnosis, and considers poor physical performance as indicative of severe sarcopenia^38^. The muscle mass was measured as the ratio of an individual’s total lean mass of legs and arms (also named Appendicular Skeletal Muscle or ASM)^39^ to their squared height (ASM/height^2^). A DXA scanner (Discovery model, manufactured by Hologic, Inc. Bedford USA: W S/N 84430) was used to measure ASM. The coefficients of variation of the DEXA for fat and lean mass were 1.5% and 0.6%, respectively. According to EWGSOP1, low muscle mass was defined as the amount of less than 5.45 (kg/m^2^) and 7.26 (kg/m^2^) for women and men, respectively^37^. These cut-off points were changed to less than 5.5 (kg/m^2^) and 7.0 (kg/m^2^) for women and men, respectively^38^. A handgrip test by a pneumatic instrument—a squeeze bulb dynamometer (manufactured by Jamar,Inc. USA: c7489-02 Rolyan) calibrated in pound per square inch (psi)—was used to scale the muscle strength. The position of the hands while doing the hand-grip strength was based on the recommendations of the American Society of Hand Therapists^40^. To do this, we asked the patient to sit in a straight-backed chair while shoulders adducted in neutral, arms unsupported and elbows flexed at 90°. At this position, forearm rotation was neutral and wrist 0°–30° dorsiflexion and 0°–15° ulnar deviated. The hand grip strength (maximum voluntary contractions) was measured three times for each right and left hand with a 30-s rest in between measurements. We used the average measurements of the participants’ both hands as their muscle strength. Sex and age-specific cutoff points suggested by Merkies et al. were used to identify low muscle strength^41^. These values were different from EWGSOP1 cutoff points, in which hand grip strength < 30 kg for men and < 20 kg for women was considered as low grip strength^37^. In the revised definition, these cut-off points were changed to < 27 kg for men and < 16 kg for women^38^. The muscle performance was measured using a 4-m walk gait speed test^37^. Participants who had gait speeds less than 0.8 m/s were identified as those with a low muscle performance^37^.

### Assessment of other variables

General information on age, sex, socio-economic status, medical history, medication use, smoking habits and alcohol consumption was collected by a trained dietitian. Physical activity level was examined by the use of a short form of International Physical Activity Questionnaire (IPAQ). The validity of IPAQ has previously been examined in elderly population^35^. Vigorous- and moderate-intensity activities and walking (for at least 10 min) were asked separately in minutes and days during the past week. Then MET scores for each activity were obtained from earlier publications^42^. The obtained MET scores were multiplied by the amount of time each participant spent on that activity, while taking into consideration the frequency of engaging in the mentioned activity during the past week. Then, the scores for different activities were summed up to obtain total MET-min/week. Weight was measured using a digital scale while participants were minimally clothed. A wall tape measure was used to assess height in standing position without shoes. Participants were asked to stand up and normally breathe to measure waist circumference at the middle of lower rib margin and iliac crest. Weight (kg) divided by height squared (m^2^) was used to calculate body mass index (BMI).

### Statistical analysis

Participants were classified based on tertiles of DASH dietary pattern scores. To compare general characteristics of study participants across tertiles of DASH dietary pattern scores, we used one-way analysis of variance and chi-squared tests, where appropriate. Dietary intakes were compared using ANCOVA, adjusting for the confounding effects of age, sex and energy intake. To find the association between DASH dietary pattern and risk of sarcopenia, we used multivariate logistic regression analysis. In the first statistical model, we controlled for age (years), sex (male/female) and energy intake (kcal/day). Further adjustment was applied for physical activity (MET-h/week), smoking (yes/no), medication use (yes/no), alcohol consumption (yes/no), and positive history of disease (yes/no) in the second model. To assess the trend of odds ratios across categories of tertiles of DASH diet score, we considered the tertile categories as an ordinal variable in the logistic regression models. All statistical analyses were conducted using SPSS, version 18 (SPSS Inc., Chicago, IL, USA). P values less than 0.05 were considered statistically significant.

## Results

General characteristics of study participants in subjects with and without sarcopenia as well as across tertiles of DASH diet score are displayed in Table 1. Patients with EWGSOP2-sarcopenia had lower BMI and diabetes and used more sexual hormone and corticosteroids than subjects without sarcopenia. Individuals in the highest tertile of DASH diet score were more likely to be female and less likely to be smoker and alcohol user, compared with those in the lowest tertile. No other significant differences were found.

**Table 1:** Characteristics of study participants in subjects with and without sarcopenia and also in tertile categories of DASH diet score.

	Sarcopenia^†^		*P*^‡^	Tertiles of DASH diet score			*P*^‡^
	Yes (n = 31)	No (n = 269)		T_1_ (n = 96)	T_2_ (n = 98)	T_3_ (n = 106)	
Age (years)	64.54 ± 6.39	67.05 ± 7.81	0.08	66.09 ± 7.81	66.50 ± 7.58	67.70 ± 7.71	0.29
BMI (kg/m^2^)	23.48 ± 2.78	27.83 ± 4.11	< 0.001	27.31 ± 4.08	27.69 ± 4.16	27.15 ± 4.37	0.65
Physical activity (MET-h/w)	1223.45 ± 1122.32	1302.72 ± 1462.33	0.77	1187.69 ± 1262.57	1357.44 ± 1606.94	1333.11 ± 1405.76	0.67
Female (%)	64.5	49.4	0.11	40.6	49.0	62.3	0.008
Alcohol use (%)	9.7	13.8	0.52	18.8	19.4	2.8	< 0.001
Smoking (%)	12.9	12.6	0.96	25.0	9.2	4.7	< 0.001
**Medical history**							
Diabetes (%)	6.5	22.3	0.03	16.7	24.5	20.8	0.40
MI (%)	6.5	12.6	0.31	12.5	11.2	12.3	0.95
CVA (%)	6.5	2.2	0.16	4.2	3.1	0.9	0.34
Arthritis (%)	3.2	1.5	0.47	2.1	2.0	0.9	0.77
Asthma (%)	3.2	1.9	0.60	2.1	1.0	2.8	0.65
**Drug history**							
Sexual hormone use (%)	9.7	2.2	0.02	2.1	2.0	4.7	0.43
Statin use (%)	35.5	36.8	0.88	33.3	38.8	37.7	0.70
ACEi use (%)^§^	3.2	8.2	0.32	7.3	7.1	8.5	0.92
Corticosteroid use (%)	12.9	1.5	< 0.001	3.1	2.0	2.8	0.88

All values are mean ± SD, unless indicated.

^†^Sarcopenia was defined based on European Working Group on Sarcopenia in Older People 2 (EWGSOP2) definition^38^.

^‡^ANOVA for continuous variables and Chi-squared test for categorical variables.

^§^ACEi: angiotensin-converting enzyme inhibitor.

Multivariable-adjusted dietary intakes of study participants across tertiles of DASH diet score are provided in Table 2. Participants in the top tertile of DASH diet score had significantly higher intakes of dairy products, fruits, vegetables, nuts, legumes and soy, dietary fiber, protein, calcium and folate and lower intakes of sugar-sweetened beverages and sweets, red and processed meats, energy, sodium and fat compared with those in the bottom tertile.

**Table 2:** Dietary intakes of study participants by tertile categories of DASH Diet score.

	Tertiles of DASH diet score			*P*^†^
	T_1_ (n = 96)	T_2_ (n = 98)	T_3_ (n = 106)	
DASH score range	< 39	39–49	> 49	
**Food groups (g/day)**				
Fruits	455.76 ± 24.07	629.17 ± 23.76	717.97 ± 22.94	< 0.001
Vegetables	404.88 ± 22.73	536.36 ± 22.44	709.17 ± 21.66	< 0.001
Nuts, legumes and soy	53.71 ± 3.70	55.64 ± 3.66	67.13 ± 3.53	0.01
Dairy products	498.26 ± 30.83	544.11 ± 30.45	628.61 ± 29.39	0.009
Grains	333.29 ± 18.09	307.94 ± 17.87	307.34 ± 17.24	0.50
Sugar-sweetened beverages and sweets	82.02 ± 7.55	29.72 ± 7.46	14.37 ± 7.20	< 0.001
Red and processed meats	54.98 ± 2.81	34.43 ± 2.77	24.92 ± 2.68	< 0.001
**Nutrients**				
Sodium	4205.86 ± 122.32	3326.08 ± 120.78	2865.86 ± 116.58	< 0.001
Energy	2396.12 ± 93.42	2044.82 ± 91.68	2343.96 ± 89.22	0.01
Carbohydrate	356.49 ± 5.61	370.58 ± 5.54	370.44 ± 5.35	0.12
Protein	83.15 ± 1.83	84.14 ± 1.80	90.47 ± 1.74	0.008
Fat	63.77 ± 1.92	58.47 ± 1.89	55.97 ± 1.83	0.01
Fiber	25.83 ± 0.81	30.21 ± 0.80	33.54 ± 0.77	< 0.001
Calcium	1223.35 ± 43.88	1332.90 ± 43.33	1449.38 ± 41.82	0.001
Pyridoxine	2.46 ± 0.13	2.60 ± 0.13	2.74 ± 0.12	0.32
Folate	486.42 ± 10.94	538.62 ± 10.80	599.84 ± 10.42	< 0.001

All values are mean ± SE; energy intake is adjusted for age and sex, all other values are adjusted for age, sex and energy intake.

^†^ANCOVA for all variables.

The prevalence of EWGSOP2-sarcopenia and its components across tertile categories of DASH diet score as well as stratified by gender are indicated in Table 3. In the whole population, muscle mass and hand grip strength were significantly lower among subjects in the highest tertile of DASH diet score compared with those in the lowest tertile. The prevalence of EWGSOP2-sarcopenia itself and its other components were not significantly different across tertiles of DASH diet score. When we did gender-stratified analyses, no significant differences were found across tertiles of DASH diet score, in both men and women.

**Table 3:** Prevalence of sarcopenia and its components across tertile categories of DASH diet score, stratified by gender.

	Tertiles of DASH diet score			P*
	T_1_	T_2_	T_3_	
DASH Score range	< 39	39–49	> 49	
**Whole population**				
n	96	98	106	
Muscle mass [ASM/h^2^](kg)	6.71 ± 1.01	6.72 ± 0.92	6.40 ± 1.01	0.02
Hand grip strength (psi)	11.82 ± 3.74	11.19 ± 3.74	10.20 ± 3.07	0.005
Gait speed (m/s)	0.86 ± 0.25	0.82 ± 0.20	0.84 ± 0.21	0.55
Lower muscle mass n (%)^†^	38 (39.6)	31 (31.6)	39 (36.8)	0.50
Lower hand grip strength n (%)^‡^	25 (26.0)	31 (31.6)	40 (37.7)	0.20
Slower gait speed (m/s) n (%)^§^	38 (39.6)	40 (40.8)	44 (41.5)	0.96
Sarcopenia n (%)^||^	11 (11.5)	7 (7.1)	13 (12.3)	0.44
**Men**				
n	57	50	40	
Muscle mass [ASM/h^2^](kg)	7.23 ± 0.74	7.20 ± 0.70	7.10 ± 0.81	0.69
Hand grip strength (psi)	13.78 ± 3.18	13.88 ± 2.94	12.89 ± 2.61	0.23
Gait speed (m/s)	0.91 ± 0.21	0.88 ± 0.18	0.87 ± 0.22	0.61
Lower muscle mass n (%)	25 (43.9)	21 (42.0)	19 (47.5)	0.87
Lower hand grip strength n (%)	9 (15.8)	9 (18.0)	7 (17.5)	0.95
Slower gait speed (m/s) n (%)	17 (29.8)	15 (30.0)	14 (35.0)	0.83
Sarcopenia n (%)	4 (7.0)	3 (6.0)	4 (10.0)	0.76
**Women**				
n	39	48	66	
Muscle mass [ASM/h^2^](kg)	5.96 ± 0.88	6.23 ± 0.85	5.97 ± 0.87	0.23
Hand grip strength (psi)	8.96 ± 2.43	8.40 ± 2.04	8.57 ± 2.00	0.46
Gait speed (m/s)	0.79 ± 0.28	0.77 ± 0.21	0.82 ± 0.20	0.51
Lower muscle mass n (%)	13 (33.3)	10 (20.8)	20 (30.3)	0.37
Lower hand grip strength n (%)	16 (41.0)	22 (45.8)	33 (50.0)	0.66
Slower gait speed (m/s) n (%)	21 (53.8)	25 (52.1)	30 (45.5)	0.65
Sarcopenia n (%)	7 (17.9)	4 (8.3)	9 (13.6)	0.41

*Obtained from ANOVA for quantitative variables and chi-square for qualitative variables (*P* < 0.05 significant).

^†^Muscle mass lower than 5.5 (kg/m^2^) for women and 7.0 (kg/m^2^) for men^38^.

^‡^Lower muscle strength were defined according previous study^41^.

^§^Gait speeds equal or slower than 0.8 m/s^38^.

^||^Sarcopenia was defined based on European Working Group on Sarcopenia in Older People 2 (EWGSOP2) definition^38^.

Multivariable-adjusted odds ratios for sarcopenia across tertile categories of DASH diet score as well as stratified by gender are provided in Table 4. We found no significant association between adherence to the DASH diet and EWGSOP2-sarcopenia (OR 1.08; 95% CI 0.45–2.54). Such finding was also seen when we adjusted the ORs for potential confounders (OR 1.04; 95% CI 0.39–2.75). The same findings were obtained in the gender-stratified analyses (men: OR 2.29; 95% CI 0.39–13.29 and women: 0.75; 95% CI 0.23–2.45). Moreover, regarding EWGSOP1-sarcopenia, we failed to find any significant association between adherence to the DASH diet and sarcopenia either before (OR 0.73; 95% CI 0.36–1.47) or after controlling for potential confounders (OR 0.78; 95% CI 0.36–1.67). Such non-significant findings were also observed in the gender-stratified analyses (men: OR 1.22; 95% CI 0.42–3.55 and women: 0.45; 95% CI 0.14–1.44).

**Table 4:** Multivariable-adjusted odds ratios (95% CIs) for sarcopenia across tertile categories of DASH diet score, stratified by gender.

	Tertiles of DASH diet score			*P* trend
	T_1_	T_2_	T_3_	
Sarcopenia*				
n	96	98	106	
Crude	1.00	0.59 (0.22–1.60)	1.08 (0.45–2.54)	0.82
Model 1^†^	1.00	0.64 (0.23–1.80)	1.06 (0.43–2.60)	0.85
Model 2^‡^	1.00	0.69 (0.23–2.04)	1.04 (0.39–2.75)	0.90
**Men**				
n	57	50	40	
Crude	1.00	0.84 (0.18–3.97)	1.47 (0.34–6.27)	0.62
Model 1^§^	1.00	0.83 (0.17–4.07)	1.71 (0.38–7.60)	0.50
Model 2	1.00	0.76 (0.12–4.72)	2.29 (0.39–13.29)	0.41
**Women**				
n	39	48	66	
Crude	1.00	0.41 (0.11–1.54)	0.72 (0.24–2.12)	0.65
Model 1^§^	1.00	0.51 (0.13–1.97)	0.79 (0.26–2.40)	0.77
Model 2	1.00	0.51 (0.12–2.16)	0.75 (0.23–2.45)	0.71
**Sarcopenia**^**||**^				
n	96	98	106	
Crude	1.00	0.64 (0.31–1.34)	0.73 (0.36–1.47)	0.38
Model 1	1.00	0.66 (0.31–1.40)	0.75 (0.36–1.55)	0.43
Model 2	1.00	0.71 (0.33–1.55)	0.78 (0.36–1.67)	0.52
**Men**				
n	57	50	40	
Crude	1.00	0.93 (0.36–2.40)	1.25 (0.48–3.25)	0.67
Model 1	1.00	0.90 (0.34–2.37)	1.06 (0.39–2.86)	0.92
Model 2	1.00	0.88 (0.31–2.43)	1.22 (0.42–3.55)	0.75
**Women**				
n	39	48	66	
Crude	1.00	0.38 (0.11–1.27)	0.46 (0.16–1.31)	0.16
Model 1	1.00	0.41 (0.12–1.38)	0.46 (0.16–1.34)	0.18
Model 2	1.00	0.42 (0.11–1.54)	0.45 (0.14–1.44)	0.20

*Sarcopenia was defined based on European Working Group on Sarcopenia in Older People 2 (EWGSOP2) definition^38^.

^†^Model 1: Adjusted for age, sex and energy intake.

^‡^Model 2: Further adjusted for physical activity, smoking, alcohol consumption, medication use (statin, ACEi, estrogen, testosterone), corticosteroid use and positive history of disease (asthma, arthritis, myocardial infarction, cerebrovascular accident, diabetes).

^§^Model 1: Adjusted for age and energy intake.

^||^Sarcopenia was defined based on European Working Group on Sarcopenia in Older People (EWGSOP) definition^37^.

Linear regression analysis of the association between DASH diet score and components of sarcopenia are presented in Table 5. In the unadjusted model, the DASH diet score was inversely related to hand grip strength. However; after adjustment for potential confounders, this association became non-significant. No other overall association was seen between DASH diet score and other components of sarcopenia.

**Table 5:** Linear regression analysis of the association between DASH diet score and components of sarcopenia.

	DASH diet score											
	Crude				Model 1*				Model 2^†^			
	β	95% CI	*P*	R^2^	β	95% CI	*P*	R^2^	β	95% CI	*P*	R^2^
Muscle mass [ASM/h^2^](kg)	− 0.006	− 0.017, 0.005	0.29	0.004	0.002	− 0.007, 0.011	0.67	0.340	0.001	− 0.009, 0.010	0.94	0.354
Hand grip strength (psi)	− 0.044	− 0.083, − 0.006	0.02	0.017	− 0.006	− 0.033, 0.020	0.62	0.571	− 0.003	− 0.030, 0.024	0.82	0.588
Gait speed (m/s)	− 0.001	− 0.004, 0.001	0.33	0.003	0.001	− 0.003, 0.002	0.85	0.112	− 0.001	− 0.002, 0.002	0.96	0.162

*Model 1: Adjusted for age, sex and energy intake.

^†^Model 2: Further adjusted for physical activity, smoking, alcohol consumption, medication use (statin, ACEi, estrogen, testosterone), corticosteroid use and positive history of disease (asthma, arthritis, myocardial infarction, cerebrovascular accident, diabetes).

Multivariable-adjusted odds ratios for EWGSOP2-sarcopenia across tertile categories of DASH diet components are shown in Table 6. In terms of vegetables, when we controlled the analysis for potential confounding variables, participants in the highest tertile were 76% less likely to have EWGSOP2-sarcopenia compared with those in the lowest tertile (OR 0.24; 95% CI 0.07–0.74). No significant associations were seen between other components of DASH diet and odds of EWGSOP2-sarcopenia, after adjustment for potential confounders.

**Table 6:** The association between components of the Dietary Approaches to Stop Hypertension (DASH) diet and sarcopenia* (Odds ratios and 95% confidence intervals)^†^

	Tertiles of components of the DASH diet			*P* trend
	T_1_	T_2_	T_3_	
Whole-grains	1.00	1.05 (0.36–3.00)	1.57 (0.59–4.15)	0.35
Fruit	1.00	0.61 (0.22–1.66)	0.80 (0.31–2.07)	0.65
Vegetables	1.00	0.57 (0.22–1.48)	0.24 (0.07–0.74)	0.01
Low-fat dairy products	1.00	2.50 (0.83–7.49)	1.88 (0.61–5.75)	0.34
Nuts, legumes and soy	1.00	0.36 (0.11–1.10)	0.75 (0.30–1.85)	0.51
Red and processed meats	1.00	1.10 (0.40–3.06)	1.19 (0.44–3.19)	0.73
Sodium	1.00	2.36 (0.87–6.36)	0.65 (0.19–2.18)	0.46
Sweats and sweetened beverages	1.00	2.11 (0.71–6.19)	1.59 (0.54–4.62)	0.43

*Sarcopenia was defined based on European Working Group on Sarcopenia in Older People 2 (EWGSOP2) definition^38^. Binary logistic regression was used to obtain OR and 95% CI. The overall trend of the OR across increasing tertiles was examined by considering the median score in each category as a continuous variable.

^†^ORs are adjusted for age, sex, energy intake, physical activity, smoking, alcohol consumption, medication use (statin, ACEi, estrogen, testosterone), corticosteroid use and positive history of disease (asthma, arthritis, myocardial infarction, cerebrovascular accident, diabetes).

## Discussion

We failed to find any significant association between adherence to the DASH diet and odds of sarcopenia after controlling for potential confounders. According to our knowledge, this is the first study from the Middle-Eastern countries that reports the association between adherence to the DASH diet and sarcopenia.

Considering increased age in the world, sarcopenia should be considered as an important public health problem^43^. Dietary intakes can play a considerable role in maintaining muscle health and strength^44^. Several studies have shown that healthy dietary patterns were inversely associated with risk of sarcopenia^45,46^. In the present study, adherence to DASH diet was not significantly associated with odds of sarcopenia. Although no previous study has examined DASH diet in relation to sarcopenia, two recent studies have reported that adherence to a diet similar to the DASH diet is not associated with sarcopenia^47,48^. A large prospective cohort study by Chan et al. among Chinese men and women aged 65 years or older revealed no significant association between Mediterranean Diet Score (MDS) and sarcopenia^48^. It should be noted that the MDS in Chan et al.’s study was based on 6 healthy components (vegetables, legumes, fruits and nuts, cereal, fish and mono-unsaturated to saturated lipids ratio) and 3 unhealthy components (meat, poultry and dairy products), which is different from the DASH diet scoring in our study. In addition, studying various populations with different dietary patterns may provide better insight into the association between adherence to a healthy dietary pattern and risk of developing sarcopenia. Findings from another prospective study on 100 outpatients aged 18 or more, with a recent diagnosis of GI cancer, revealed that adherence to a diet rich in legumes, vegetables and fruit was not significantly associated with sarcopenia^47^. However, it must be kept in mind that this study has been done on patients with cancer, in which cytokines can lead to severe muscle wasting (sarcopenia) and cachexia^49^. Therefore, this pattern of sarcopenia might be different from the one we studied in the current study and their findings cannot easily be generalized to other healthy populations. Unlike our findings, a cross-sectional study indicated a favorable association between adherence to the DASH diet and handgrip strength, as a component of sarcopenia^32^. In a quasi-experimental study, combined resistance training (RT) along with a counseling on modified DASH diet in 95 overweight and obese older adults aged 55 to 80 years resulted in significant improvements in muscle strength, as measured by handgrip strength and knee extensor torque^33^. Lack of any significant association between adherence to DASH-style diet and sarcopenia might be partially explained by the low number of sarcopenic patients in our study. When we categorized participants based on tertiles of DASH diet score, only 18 participants in the top tertile had sarcopenia. Such a low number of patients would result in a very wide range of confidence intervals, which can hide the probable association of DASH diet and sarcopenia. In addition, we used the squeeze bulb dynamometer for hand grip strength instead of the Jamar handheld dynamometer, which has been accepted as the gold standard^50^. Therefore, some sort of misclassifications of study participants in terms of sarcopenia cannot be ignored. Further studies with a large sample of sarcopenic patients might be required to shed light on this association.

We did not find any significant association between adherence to DASH diet and odds of sarcopenia. However, there are some probable mechanisms that can help explaining the association between DASH and sarcopenia. Consumption of DASH diet results in improving poor nutritional status, suppressing inflammation process^51^, reducing oxidative stress as well as neutralizing mild metabolic acidosis^16,52–54^. These pathways may help preventing age-related muscle mass and strength decline.

This study has several strengths. This is the first report examining the association between adherence to the DASH diet and risk of sarcopenia. We controlled for a wide range of confounders in the current analysis. In addition, we used a validated FFQ for assessment of dietary intake. Our study has some limitations. The major one is its cross-sectional design, which precludes us inferring causal associations. Thus, our findings need to be confirmed in prospective studies. Due to the use of the FFQ, misclassification of study participants and measurement error are another concerns. In addition, the FFQ could not provide precise measurement of sodium intake while it is a major component of the DASH diet. Also, we did not collect 24-h urine samples of participants to assess sodium intake. Moreover, due to financial constraints as well as limited access to the only available DEXA device in Tehran (maximum 300 cases), this study was done on a small sample from the confined region where the device was located. Therefore, caution is needed when generalizing the results to the whole Iranian population. Further, we had only collected information on arthritis among musculoskeletal diseases. No information was collected about other musculoskeletal disease and anti-psychotic drugs in the current study. Finally, our study consisted of a relative young population including 300 subjects aged ≥ 55 years, which can explain the low prevalence of sarcopenic subjects in the sample enrolled.

In conclusion, we found that adherence to the DASH diet was not significantly associated with odds of sarcopenia after controlling for confounders. Future prospective studies are required to confirm these findings.
